# Stakeholders perspectives of barriers and facilitators of childhood obesity prevention policies in Iran: A Delphi method study

**DOI:** 10.1186/s12889-021-12282-7

**Published:** 2021-12-11

**Authors:** Shahnaz Taghizadeh, Mahdieh Abbasalizad Farhangi, Rahim Khodayari-Zarnaq

**Affiliations:** 1 Department of Community Nutrition, Tabriz University of Medical Sciences, Tabriz, Iran; 2 Department of Health Policy and Management, School of Management and Medical Informatics, Tabriz University of Medical Sciences, 14711, Tabriz, 5166614711 Iran

**Keywords:** Barriers, facilitators, childhood obesity, prevention, policy, Iran

## Abstract

**Background:**

The prevalence of obesity among children and adolescents is one of the most important health challenges of the present century. Many factors affect the prevention policies related to this health problem and make their implementation difficult. This study examined perceived barriers and facilitators of childhood obesity prevention policies by stakeholders.

**Methods:**

A qualitative descriptive research design based on Delphi method was conducted. In addition, semi-structured one-to-one interviews were conducted with childhood obesity prevention policy stakeholders (n=39) and initial identification of barriers and facilitators in this area. Interviews were digitally recorded, transcribed verbatim, and finally analyzed, followed by using thematic analysis. Subsequently, two-round Delphi panel was done by sending e-mails to stakeholders (21 stakeholders participated in the first round and 15 stakeholders in the second round) for the final selection of barriers and facilitators of obesity prevention policies among children and adolescents in Iran.

**Results:**

The identified barriers and facilitators were divided into three levels: individual, executive, and structural. Barriers and facilitators of the structural level showed a high score and priority regarding obesity prevention policies among children and adolescents.

**Conclusion:**

The existence of significant barriers at all three levels and especially at the structural level were among the concerns of stakeholders.

**Supplementary Information:**

The online version contains supplementary material available at 10.1186/s12889-021-12282-7.

## Background

The increasing prevalence of obesity among children and adolescents is one of the most important health challenges of the 21^st^ century leading to serious health problems [1, 2]. Obesity is a complex disease in all age groups, and the imbalance between energy intake and energy expenditure is recognized as its main development mechanism [3]. According to the statistics released by the United Nations Children's Fund (UNICEF) in 2016, one in five children and adolescents are obese and overweight [4]. Studies show that the prevalence of overweight and obesity in developing countries is 30% higher than in developed countries [5].

Iran is one of the developing countries with a high prevalence of childhood obesity. A study carried out in Iran in 2017 showed that about 14% of children under five years of age were overweight and obese [6]. Moreover, the results of Childhood and Adolescence Surveillance and Prevention on of Adult Non-communicable disease (CASPIAN-V) study conducted on 14,274 Iranian students from 31 provinces in the academic year 2014-2015 showed that in the age groups of 7-10 years, 11-14 years, and 15-18 years, 11.1%, 12.2%, and 10.8% of children and adolescents were obese, respectively [7, 8].

Through a macro level analysis of the prevalence of obesity among all age groups, including children and adolescents, various reasons can be identified at the community level making a person exposed to high energy intake or sedentary lifestyle. Given that the high prevalence of obesity and non-communicable diseases can cause huge costs to the public health system, and at the same time, have irreversible adverse effects on the health system and society [9, 10], it seems that the existence of obesity-related policies in any society can have a great impact on food intake or physical activity of people in that society. In 2010, the World Health Organization (WHO) estimated that only about 43% of the world's countries have some predefined strategies, policies, or programs to control obesity, of which 60% are European countries and 64% are high-income ones [11]. Despite efforts to prevent childhood obesity, the prevalence of obesity among this age group in Iran is still high. However, there is no comprehensive study investigating the prevention policies of childhood obesity in Iran. In addition, no study has evaluated the effects of barriers and facilitators influencing childhood and adolescent obesity prevention policies in the obesity process in Iran so far. Therefore, this study was designed with the aim of identifying the barriers and facilitators affecting children and adolescent obesity prevention policies in Iran in order to provide new insights for managers, planners, consultants, decision makers, and policy makers. It is also hoped that this study can pave the way for other researchers to conduct other applied studies to effectively address this health challenge.

## Methods

This study was conducted adhering to a qualitative-descriptive research design which was part of a large-scale research entitled " Futures study and policy analysis of the prevention of obesity in children and adolescents in Iran and providing policy options ". Accordingly, after reviewing the related documents, websites, and literature and following the instructions made by three key stakeholders in Iran, a list of key informants was prepared. These three stakeholders in policy-making. One of these stakeholders was national and one provincial level policymaker from the Ministry of Health, Treatment and Medical Education who were very familiar with the individuals and organizations involved in this policy. Another stakeholder was university professor at the provincial level because of her work experience and familiarity with organizations that cooperate in the policy.

There were no relationships with participants prior to study. Key informants were identified as targeted sampling method. Telephone or in-person arrangements were made for the interview time. Then, after making using a semi-structured interview and a snowball sampling method, 39 key informants, after informing them of the objectives of the study, were interviewed in the workplace using interview guide (interview guide supplementary file 2). When most of the answers were received repeatedly from stakeholders, it indicated that reaching the saturation phase and we ended the interviews.

The interviewer tried to avoid any bias or prejudice during the interview. Field notes were made during the interview and to complete the text of the interview, participants were asked to record their voices during the interview if they were satisfied. transcripts returned to participants for comment and/or correction. A tentative framework for barriers and facilitators of childhood and adolescent (0-18 years old) obesity prevention policies was developed. The comments from the semi-structured interviews were coded by researcher, the obtained codes were classified together according to their similarity to form themes and sub-themes. Themes divided into three levels: individual, executive, and structural levels. The levels constructed from the expert reviews were formed according to the Theoretical Domains Framework (TDF) [12].

Using the themes and the results of our previous systematic review [13], a preliminary list of barriers and facilitators of childhood and adolescent obesity prevention policies was obtained, and its validity was confirmed by five experts. The acceptable proposed barriers and facilitators had a strong and provable retrospective effect.

After initial in-person or telephone arrangements to participate in the Delphi panel and complete the demographic profile during these arrangements, a 10-point Likert scale containing the initial questions of identifying the barriers and facilitators was forwarded to 21 key informants via e-mail. In this questionnaire, which was provided as a supplementary file 1, the highest score was assigned to a barrier or facilitators in the structure of Iranian society with a high impact on childhood and adolescent obesity prevention policies, and lower scores were assigned to the barriers or facilitators identified as less important by the participants. Furthermore, the participants were free to leave their comments on each of the statements, and to add any new barriers or implications. At this stage, the questions with an average score of 1-3 were removed from the selected items for the second round, and the questions with an average score of 4-6 entered the second round of the Delphi panel for prioritization by the key informants. In the second round, the participants were asked to rate the remaining questions and assigned their intended scores to them. Questions with a score above 6 entered the selected priorities. In this study, Standards for Reporting Qualitative Research (SRQR) were employed in describing the design and findings [14]. There are several definitions of childhood obesity. In this study, according to WHO criteria, for children lees than 5 years old, weight for height, more than 3 SD and from 5 to 19 years old, BMI for age, more than 2 SD are considered as obesity children or adolescents [15].

## Positioning and characteristics of the researchers

The research team in this study comprised of two postgraduate qualified researchers and one Ph.D. student from Tabriz University of Medical Sciences. All researchers had received education in related research techniques.

## Data Analysis

Stata software version 12 was used to summarize the general characteristics of the participants. Using MAQDA10 software, the data obtained from the interviews and Delphi panels were analyzed by thematic analysis.

## Trustworthiness

Lincoln and Guba’s criteria [16] for trustworthiness (i.e., transferability, credibility, confirmability, and dependability) were used to boost the trustworthiness of this study. In addition, the transcripts and the translated texts were read by the researchers’ various times to enhance the credibility and dependability of the data. Subsequently, the transcriptions were notified to the participants. Furthermore, participants’ quotes were presented verbatim to expand data transferability. The themes were then constructed through conformability by the research team. Other measures for ascertaining trustworthiness were concurrent analysis to obtain a full understanding of themes, the authors’ discussion about the emerged themes, and periodical coding reviews, and classification and analysis of barriers and facilitators ranked by stakeholders.

## Results

The in-depth semi-structured interviews with stakeholders were conducted by the lead researcher. Out of 39 interviews conducted from February 2019 to June 2020, 34 cases were conducted in person and 5 cases were conducted by phone calls due to the Coronavirus disease 2019 (COVID-19) pandemic. Of the 39 stakeholders participating in the interview phase, 9 participants agreed to enter the Delphi panel. Afterwards, 12 other stakeholders were added, making the number of participants 21 in the first round of the Delphi panel; meanwhile, 15 stakeholders participated in the second round of the Delphi panel. The duration of this study was 9 months (interviews 4 months and Delphi panel 5 months). Data were analyzed by thematic analysis in five stages as follows:

1. The initial statements from the interviews were classified as themes.
2. The themes were divided into two groups: barriers and facilitators.
3. To determine the relative importance of each factor, the assigned Likert scores were calculated as average scores.
4. Barriers and facilitators were finalized and divided into three levels: individual, executive, and structural.
5. The existing barriers and facilitators were prioritized and analyzed separately.

Table 1 Shows the organizational characteristics of the participants in the semi-structured interview phase and rounds 1 and 2 of Delphi panel. Table 2, also, shows the demographic characteristics of the participants.

**Table 1:** Organizational characteristics of the participants

Participating organization	Subset of participating organization	Interview (n=39)	1st Stage of Delphi (n=21)	2nd Stage of Delphi(n=15)
Ministry of Health and Medical Education (MoHME)	Health Deputy of MoHME*	1	1	1
	Professor of university of medical sciences	2 ^a^	7 ^b^	3 ^c^
	Department of School Health	1	-	-
	Office of Community Nutrition Improvement	2	3	3
	Department of Population and Family Health of Provincial Health Center	4	-	-
	Department of Community Nutrition Improvement of Provincial Health Center	1	3	3
	Department of Non-Communicable Diseases of the Provincial Health Center	1	-	-
	School Health Department of the Provincial Health Center	1	1	-
	Health care Providers of Provincial Health Center	1	1	1
	Secretariat of the Health and Food Safety	1	-	-
	Food and Drug Organization	1	-	-
	Provincial Food and Drug Office	1	-	-
Ministry of Science, Research and Technology	Professor of the Faculty of Agriculture	-	1	1
Welfare Organization	Provincial Welfare Organization	1	-	-
	Welfare Organization	1	1	1
Ministry of Education	Executive Manager of MoE	1	-	-
	Provincial Executive Manager	4	1	1
		7	1	1
NGO	Student Organization	1	-	-
Private sector	Managing Director of Food Production Factory	1	-	-
Children and Adolescents' Intellectual Development Center	Provincial Children and Adolescents' Intellectual Development Center	1	-	-
Municipality	Municipality of Tabriz	1	1	-
Islamic Republic of Iran Broadcasting (IRIB)	Islamic Republic of Iran Broadcasting (IRIB)	1	-	-
Ministry of Sports and Youth	Ministry of Sports and Youth	1	-	-
Imam Khomeini Relief Committee (R)	Provincial Imam Khomeini Relief Committee	1	-	-
Islamic Development Organization	Islamic Development Organization	1	-	-

^a^(1 nutritionist, 1 pediatrician), ^b^ (1 nutritionist, health policy specialist, 1 health economics, 1 social medicine specialist, 2 pediatricians and 1 food and nutrition policy specialist), ^c^(1 nutritionist, 1 health policy specialist, 1 pediatrician

**Table 2:** Demographic characteristics of the participants

Characteristics	Interview (n=43)	1st Stage of Delphi (n=21)	2nd Stage of Delphi(n=15)
Sex	18 M, 21 F*	8 M, 13 F	7 M, 8 F
Work experience (mean)	15.47	13	13.6
Age(mean)	44.76	43.61	43.26

*Female

The results of the Delphi panel are shown in Tables 3 and 4. In the second round, the factors that gained a score of less than 3 in the first round, were removed and the titles of some barriers and facilitators were corrected and some factors were added to the list of the first round by participants. The results of the second round are thematically analyzed and divided into three levels: individual, executive, and structural. Finally, 45 barriers (11 at the individual level, 12 at the executive level, and 22 at the structural level) (Table 3) and 20 facilitators (1 at the individual level, 11 at the executive level, and 8 at the structural level) (Table 4) were identified by the experts. Barriers of the structural level (score: 7.41) and facilitators of the structural level (score: 7.64) had the highest score among all other levels of barriers and facilitators.

**Table 3:** Barriers to childhood obesity prevention policies in Iran

Barriers level	Barriers	score
Individual (children, adolescents and parents (n = 11 )	Lack of sufficient knowledge and risk perception in children and adolescents	8.33
	Lack of active transport by parents	8.33
	Lack of sufficient knowledge in children	8.24
	Lack of Self-regulation and self-control when eating in children	8.14
	Misunderstanding of weight status by parents ^a^	7.29
	Parents' financial problems	6.8
	Misunderstanding of weight status by children ^b^	6.47
	Parent’s reluctance to become involved in COP activities and poor utilization of maternal and child health services by parents ^c^	6.16
	High family income	5.87
	Eating disorders (eg, bulimia nervosa) in children and adolescents	5.36
	lack of time and high academic pressures in children and adolescents	3.83
	**Mean points**	**6.80**
Executive (n = 12)	Lack of proper monitoring and control of policies announced for implementation	7.2
	Insufficient cooperation of stakeholders	7
	Lack of skills in communicating with school health educators or health care providers with children, adolescents and parents	6.92
	Insufficient knowledge of school health educators and health care providers	6.78
	Lack of communication skills in school health educators with children and adolescents	6.77
	Lack of commitment of schools in implementing intervention programs	6.71
	Lack of sufficient time and opportunity for executives to implement policies properly	6.39
	Insufficient cooperation of school health educators with other health care providers	6.35
	Lack of clarity of strategies and policy guidelines communicated for implementation	6.29
	Lack of peace of mind of executive staff (for example, school officials and health care providers) to carry out interventions	5.79
	High workload of teachers or health care providers	5.69
	Frequent changes in the workplace of teachers and health workers	5.54
	**Mean points**	**6.45**
Structural (n=22)	Unsafe and unsuitable physical activity environments for children and adolescents (on the streets, parks and sports clubs)	8.27
	Lack of proper transportation plans	8.25
	High access of children and adolescents to unhealthy food	8.13
	Lack of equipment and facilities ^d^	8.11
	Obegenic environments in family, schools and community	8
	Cultural problems of sports for girls such as cycling and ....	7.95
	Existence of incorrect and unscientific information of childhood obesity in society	7.95
	Insufficient commitment at management and executive levels	7.79
	Widespread advertising of fast food (poor- nutrient and high-energy foods)	7.77
	Infrastructure problems near schools, such as the abundance of fast food stores near schools	7.74
	Lack of data on the effectiveness of childhood obesity policies	7.62
	Lack of mandatory weight control for all school students	7.43
	Restrictive policies ^e^	7.39
	Allocation of subsidies or lack of taxes for unhealthy food	7.17
	Lack of space for preventive interventions such as exercise	7.1
	Problems with agenda setting) Prioritize politics(	7
	Inadequate time to provide preventive services to children, adolescents and parents ^f^	6.77
	Top-down process planning and implementation approach	6.67
	Lack of manpower	6.65
	Emergence of other unforeseen immediate priorities other than obesity in the community and school	6.5
	Legal barriers (such as administrative bureaucracies) to intervention programs	6.5
	Inconsistent policies in preschools and schools ^g^	6.3
	**Mean points**	**7.41**

^a^for example, overweight and obese children and adolescents are considered normal weight

^b^for example, overweight and obese children and adolescents consider themselves normal weight

^c^It may be related to awareness, cultural, economic, psychological and other factors.

^d^such as lack of sufficient financial resources to provide free school meals, adequate sports or educational facilities

^e^policies that limit the implementation of childhood obesity prevention policies. for example, the policy of subsidizing the import of sugar

^f^for example, interfering the policy intervention with students' school hours as well as not providing services other than working hours for working parents)

^g^For example, it is recommended to increase physical activity on the one hand and increase the academic pressures in schools and allocating physical activity times to other lessons on the other hand

**Table 4:** Facilitators of childhood obesity prevention policies in Iran

Facilitators level	Facilitators	score
Individual (children, adolescents and parents (n = 1)	Provide intervention components that are popular and desired by children and adolescents ^a^	**7.21**
Executive (n = 11)	Good relationships and teamwork between parents and school staff	7.61
	Participatory approach between stakeholders to develop the components of the intervention program	7.51
	Use of obesity-related messages for clients (e.g. distributing brochures, videos, and educational pamphlets)	7.46
	Using obesity-related messages to the general public through campaigns and advertisements, etc.	7.46
	Existence of strong motivation in teachers	7.4
	Commitment of schools	7.35
	Effective communication between executive stakeholders	7.11
	Use of obesity-related messages for policymakers (informing policy makers by health professionals)	7
	Existence of formal and informal leaders in the programs	6.56
	Minimize employee workload	6.24
	Use of experts from international organizations (when developing, planning and guiding the implementation of the program)	4.71
	**Total points**	**6.94**
Structural (n=8)	Provide a healthy eating plan in schools	8.22
	Integration of intervention programs with the curriculum in the schools	8.10
	Flexibility of intervention programs	7.90
	Availability of suitable facilities (financial and manpower)	7.74
	Full Supporting of intervention programs	8.44
	Proper monitoring and control of policies announced for implementation	7.27
	Introduction and gradual implementation of intervention programs	7.16
	Existence of sufficient executive staff	6.33
	**Total points**	**7.64**

^a^In this study, according to experts, only one Facilitator was identified at the individual level

### Barriers of the Individual Level

This level of barriers, which refers to barriers related to children, adolescents, and their parents, with an average score of 6.80, ranked second among the three levels of barriers. In this study, insufficient knowledge of parents and children and lack of individual’s awareness of body weight status were identified as the most important barriers related to the individual level. As it turns out, most of the barriers are related to low awareness and understanding at the individual level as well as financial and economic issues. Among all individual level barriers, six barriers were related to parents and five of them were related to children and adolescents. As shown in the table, just as the existence of low family income was one of the economic barrier in the implementation of this policy, high family income was also, recognized by some stakeholders as a barrier, but the score of low income were more than the high income situation (6.8 vs 5.87).

### Barriers of the Executive Level

Most of the executive barriers, which ranked third among the three levels, were school-related barriers. Barriers such as inadequate monitoring, awareness and skills, as well as insufficient cooperation between stakeholders scored the most and the important barrier selected by the participants.

### Barriers of Structural Level

Barriers of structural level (score: 7.41) were identified as the most important level, and they had the highest score among the selected barriers. Of all the structural level barriers, five were directly related to physical activity problems and three were related to unhealthy foods consumption, but most of these cited as related barriers in in policy-making and agenda setting.

### Facilitators of Individual Level

As can be seen in Tables 3 and 4, in general, the barriers were more than the facilitators in this study. Moreover, at the individual level, those interventions that were desirable and popular among children and adolescents (score: 7.21) were the only facilitators that were selected by stakeholders as important and effective in the prevention of obesity among children and adolescents.

### Facilitators of Executive Level

Facilitators of executive level (score: 6.94) ranked third among all facilitators. Favorable cooperation between stakeholders as well as increasing awareness at different levels by giving or sending messages to different target groups were important facilitators of the executive level.

### Facilitators of Structural Level

Similar to the barriers of structural level, the facilitators of structural level had the highest score (7.64) among the three levels of facilitators. Structural level facilitators address issues that are often related to policy formulation, implementation, and monitoring. Despite the fact that the number of these facilitators is lower than the number of executive levels, but due to their importance in the childhood obesity prevention policies, they have a high score compared to the executive levels.

## Discussion

This study identified the main barriers and facilitators involved in childhood and adolescent obesity prevention policies in Iran. As far as the researchers investigated, this is the first study to evaluate the barriers and facilitators of the mentioned policies from the perspective of the main policy makers of Iran. In addition, our study attempted to fill the existing gap through introducing the factors affecting the implementation of interventions related to these policies.

The most important type of barriers to the implementation of childhood and adolescent obesity prevention policies was structural level barriers. In this regard, the insecurity of physical activity environments for children and adolescents was one of the structural barriers. Physical activity in children and adolescents, as both professional and recreational sports, requires a safe environment where parents and children feel less at risk. One of the most important factors in this regard is the security of roads to/from schools. Heath *et al.* showed that environmental policies and interventions to improve the safety of physical activity environments, such as building safe cycling routes, significantly increase the level of physical activity in children and adolescents [17].

Similar to our study, the results of the determinants of diet and physical activity (DEDIPAC) study, which examined the barriers to physical activity and food intake in the school environment through interviewing key stakeholders, showed that insecurity in physical activity environments can be a barrier to the level of physical activity in children and adolescents [18]. Among the important structural barriers was the high access of children and adolescents to unhealthy foods. Selling unhealthy foods in the community, especially in stores near the schools and even inside them, often at low prices, can increase the access of children and adolescents to such foods and pose health risks [19]. Studies show that the proximity of such stores to schools, for example less than 200 meters in the study by Caraher [20] or less than 800 meters in the study by Davis [21], can change the eating patterns of students and result in obesity among them. Furthermore, taxing unhealthy foods [22, 23], subsidizing some healthy foods [22, 24], and using food guide labels on the packages [25] are among the main recommended strategies. Other suggested strategies to prevent obesity among children and adolescents include: teaching nutritional facts to children and adolescents, holding workshops for parents, supporting intervention policies [26–28], and running social health-related campaigns. For example, a campaign entitled "Say No To Fast Food" was run in Iran in 2017.

Extensive advertisement of fast foods, as one of the most effective factors in increasing obesogenic environments, was selected as another barrier in the implementation of childhood and adolescent obesity prevention policies, Similar to the results of Cyril *et al.* (2017) study [29]. More than 60% of television commercials in Iran are related to food products [30] , and most of these commercials advertise unhealthy foods that strongly affect children's food choices [31]. In this regard, in 2009, advertising unhealthy foods was restricted in the United Kingdom; as a result, children aged 3-9 years and 10-15 years were 52% and 22% less exposed to these commercials, respectively [32].

Some studies reported the following individual barriers: insufficient awareness and lack of understanding the risk of obesity, lack of self-control when eating, and high stress in students [33]. However, as the results of the present study and several other studies [34] indicated, desirable and popular interventions could solve the above problems to some extent and serve as a facilitators for childhood and adolescent obesity prevention policies. Although the overlap of intervention programs with students' school hours was cited as a barrier to implement the policies, one of the most important structural facilitators in this study, similar to the study by Hayes *et al.*, was the integration of intervention programs with the school curriculum. In a different study, Villegas *et al.* claimed that if we can incorporate the policy interventions into the school curriculum by offering more variety in the relevant subjects as well as by the behavioral management of school staff and the timely provision of teaching resources, we can increase awareness among the staff and students and implement the health policies, such as preventing childhood obesity, in a much better way [35].

Lack of comprehensive data on the effectiveness of childhood obesity prevention policies and lack of mandatory weight control for all school students were identified as other barriers. In some countries the effectiveness of childhood and adolescent obesity prevention policies has been measured by different methods [36–38], but there is no such data on the effectiveness of these policies in Iran, leading to problems in policymaking. Having data on effectiveness and knowing which methods have had the greatest health benefits for society make it easier for policymakers to decide on the type and method of policies. These analyses are important because the financial resources and staffing of the health system are limited. Hence, in a world with limited resources, it is expected that effective interventions with the greatest benefits be selected [39].

The impact of parents on childhood and adolescent obesity prevention policies has been studied in various studies, and it has been shown that parental involvement has a key role [28, 36]. In this regard, the results of the present study were in line with the studies that showed insufficient knowledge and awareness [29], difficulty in the effective use of children's health services [40], improper use of vehicles [18], and lack of participation in intervention programs [26] are some important barriers in advancing the childhood obesity prevention policies. Therefore, through appropriate planning and policies, and especially designing programs to increase parental awareness and attitude towards the complications of childhood obesity and methods to prevent it, these barriers can partially be solved. Similar to our study, other studies identified lack of staffing [28], financial constraints at the implementation level [26, 41], and at the family level [33, 42] as the barriers to this policy.

There are many barriers to access and consume a healthy diet, even when people prefer to eat healthy foods. This problem is especially important for the lower socio-economic groups. Many of these barriers are due to the structure of a society's food system [43]. Studies show that healthy foods are usually more expensive than less healthy foods [44], and that low-income families cannot afford healthy and culturally appropriate diets [45]. In the United States, a study examining school nutrition programs showed that funding from the government and private institutions can help in shaping the effective policies, and it can lead to positive changes in food and beverages sold in the school environments [46].

The selected approach in policymaking was identified as another effective factor in childhood and adolescent obesity prevention policies. The findings of the present study showed that due to the existence of a top-down policy approach, the implementation of most policies encounters serious problems. Other studies have shown that the centralized healthcare system in Iran has resulted in top-down policies, as well as implementation problems [47]. In top-down policies, there is not much consultation with executive stakeholders in the systems, and policymakers announce the policies to lower levels based on their own experiences. In such an approach, the desired goals are often not met; it may also destroy the innovations and policies developed [48].

Insufficient knowledge and communication skills along with frequent transfers of the employees are among the main barriers related to the executive staff [49]. Since the Iranian healthcare system has undergone fundamental changes in recent years, it is believed that with the addition of executive processes and assigning new tasks to the employees, many functional changes are made in the system [50]. In addition, the knowledge, skills, and practices needed in one area may be different from another one. Similar to some other studies, another barrier in the present study was the lack of sufficient cooperation between stakeholders [47]. According to Adhikari, unless there is sufficient cooperation between stakeholders, we should not expect effective implementation of health policies in that system [51].

Nowadays, stakeholders at the policy formulation and implementation level, have a high level of ability to influence childhood and adolescent obesity prevention policies. Therefore, their participation in the process of policy making can play an important role in reducing the prevalence of childhood and adolescence obesity. In addition, these stakeholders should be aware of the barriers and facilitators of this policy and manage them to effective participation in this policy. Based on the findings, nurse leaders should make the best use of the window of opportunity for involvement in the policy-making process. In addition, The Ministry of Health, Treatment and Medical Education, as the main trustee of childhood and adolescent obesity prevention policies in Iran, should have the training, support and opportunity for involvement other stakeholders with effective advocacy.

## Strengths and Limitations of the Study

Since in the present study, the views of the main stakeholders and national policymakers in the field of prevention of childhood and adolescent obesity were considered, our results can be used for policymaking at the national level. One of the main limitations of the present study was the lack of cooperation of some stakeholders due to the COVID-19 pandemic, which is expected to be overcome in future studies by other researchers. Another limitation of this study was that this study focused on stakeholders only, not on end users, and thus the results can be used more by stakeholders in policy making and policy implementation.

## Conclusion

Identifying the most important barriers and facilitators of childhood and adolescent obesity prevention policies provides important key points in implementing these policies. Since the main focus of public health system is currently on changing individual behavior, and to some extent, changing the structure of society, if the identified facilitators are not supported and the related barriers are not handled properly, we cannot expect community level aims to be achieved. Given that the complex relationships between environment, culture, food systems, and health behaviors were highlighted in this study, the identified barriers and facilitators could provide a new approach to the country's decision makers and policy makers.

## Supplementary Information

**Additional file 1.**

**Additional file 2.**

**Additional file 3.**

**Additional file 4.**

## Data Availability

The datasets used and/or analyzed during the current study are available from the corresponding author on reasonable request

## Abbreviations

CASPIAN: Childhood and Adolescence Surveillance and Prevention of Adult Non-Communicable Disease
UNICEF: United Nations Children's Fund
SRQR: Standards for Reporting Qualitative Research
COVID-19: Coronavirus disease 2019, WHO: World Health Organization
