# Supplementary material for: Stakeholders perspectives of barriers and facilitators of childhood obesity prevention policies in Iran: A Delphi method study
Source: BMC Public Health. 2021 Dec 11;21:2260. doi: 10.1186/s12889-021-12282-7 (PMC8665716; doi:10.1186/s12889-021-12282-7)
Supplement: Supplementary file 1 — Additional file 1. [file 12889_2021_12282_MOESM1_ESM.pdf]

AVA

AVA LANGUAGE CENTER

December 25, 2020
To Whom It May Concern,

Hereby it is confirmed that the paper entitled "**Barriers and facilitators of childhood obesity prevention policies in Iran: A Delphi method study**" by *Rahim Khodayari-Zarnaq* has been copy-edited by an editor of this center.

It seems that the paper enjoys a good quality in terms of writing, syntax, and semantics. The center cannot comment on the contents of the paper or anything else related to the topic of the study.

Sincerely,
Amir Shojaei
Supervisor of Ava Language Center
Tel: +989379809313 / avalc@yahoo.com
