# Supplementary material for: Stakeholders perspectives of barriers and facilitators of childhood obesity prevention policies in Iran: A Delphi method study
Source: BMC Public Health. 2021 Dec 11;21:2260. doi: 10.1186/s12889-021-12282-7 (PMC8665716; doi:10.1186/s12889-021-12282-7)
Supplement: Supplementary file 2 — Additional file 2. [file 12889_2021_12282_MOESM2_ESM.docx]

Supplementary file 1.

Dear expert

The present questionnaire is conducted in the direction of research entitled " Futures study and policy analysis of the prevention of obesity in children and adolescents in Iran and providing policy options " which is a thesis on food and nutrition policy under the guidance of Dr. Mahdieh Abbasalizad Farhangi. With your cooperation, we intend to identify and rank the barriers and facilitators affecting children and adolescent obesity prevention policies in Iran. We hope that the results of this study will be used in health policies in Iran. Primary barriers and facilitators affecting childhood and adolescent (0-18 years old) obesity prevention policies were extracted and categorized through systematic review of various studies and semi-structured interview by several experts. In this regard, in order to determine the final barriers and facilitators and the degree of importance / potential of impact and uncertainty of barriers and facilitators, taking the context of our country, this primary list of barriers and facilitators as a questionnaire is presented in Tables 1 and 2 and will be sent to you. Your and other stakeholders selected items will be summarized and will be used for finalization in the second stage. A summary of the findings will also be sent to you at the end of the study. Therefore, please complete it accurately by allocating enough time. Thank you for your sincere cooperation. It should be noted that your participation in the study and completing the questionnaire is completely voluntary and you are free to cancel your participation at any time. In addition, the information contained in the questionnaire is completely confidential and your cooperation in this matter means your informed consent to participate in this research. your participation in this research will be very valuable and promote community health. Please comment after reading the following suggested barriers and facilitators. Please consider your answer in this regard in the Likert scale with the lowest rate (1) and the highest rate (10). Also, any barriers and facilitators that are not included in these tables and seem important to you, please indicate them in the desired location.

Please express your satisfaction by checking the ✓ in this box

If you have any questions or issues, you can contact me via e-mail sh.taghizadeh54@gmail.com

Thank you for your cooperation

Shahnaz Taghizadeh

PhD student in Food and Nutrition Policy, Tabriz University of Medical Sciences

| **Table 1.** Barriers of childhood obesity prevention policies | | | | | | | | | | | | |
| --- | --- | --- | --- | --- | --- | --- | --- | --- | --- | --- | --- | --- |
| **Themes** | **Barriers** | 1 | 2 | 3 | 4 | 5 | 6 | 7 | 8 | 9 | 10 | **Any comment** |
| **Individual level *** | Insufficient risk perception in children and adolescents |  |  |  |  |  |  |  |  |  |  |  |
|  | Insufficient self-regulation and self-control when eating in children and adolescents |  |  |  |  |  |  |  |  |  |  |  |
|  | Insufficient coping skills in children and adolescents |  |  |  |  |  |  |  |  |  |  |  |
|  | Insufficient time in children and adolescents |  |  |  |  |  |  |  |  |  |  |  |
|  | Language problem ^a^ |  |  |  |  |  |  |  |  |  |  |  |
|  | Insufficient knowledge in parents |  |  |  |  |  |  |  |  |  |  |  |
|  | Difficulties in health information utilization among parents |  |  |  |  |  |  |  |  |  |  |  |
|  | Poor utilization of maternal and child health services by parents |  |  |  |  |  |  |  |  |  |  |  |
|  | Lack of active transport by parents |  |  |  |  |  |  |  |  |  |  |  |
|  | Parents' financial problems |  |  |  |  |  |  |  |  |  |  |  |
|  | Parent’s reluctance to become involved in COP activities |  |  |  |  |  |  |  |  |  |  |  |
|  | Difficulty accessing childhood obesity prevention programs for parents |  |  |  |  |  |  |  |  |  |  |  |
|  | ***What you need to add to the list above*** |  |  |  |  |  |  |  |  |  |  |  |
| **Executive level** | Insufficient cooperation of school health care providers with other healthcare providers |  |  |  |  |  |  |  |  |  |  |  |
|  | Insufficient confidence in health care providers communication skills |  |  |  |  |  |  |  |  |  |  |  |
|  | Insufficient knowledge in health care providers |  |  |  |  |  |  |  |  |  |  |  |
|  | Time limitations in stakeholders in the implementation level |  |  |  |  |  |  |  |  |  |  |  |
|  | Nurses concerns about discuss of obesity stigma as a barrier to the parents |  |  |  |  |  |  |  |  |  |  |  |
|  | High teacher or health care providers workload |  |  |  |  |  |  |  |  |  |  |  |
|  | Teamwork |  |  |  |  |  |  |  |  |  |  |  |
|  | Insufficient sense of responsibility in assistant cooks |  |  |  |  |  |  |  |  |  |  |  |
|  | Insufficient knowledge in assistant cooks |  |  |  |  |  |  |  |  |  |  |  |
|  | Passive managerial commitment |  |  |  |  |  |  |  |  |  |  |  |
|  | Existence of misinformation in the society |  |  |  |  |  |  |  |  |  |  |  |
|  | ***What you need to add to the list above*** |  |  |  |  |  |  |  |  |  |  |  |
| **Structural level** | Insufficient space in the building |  |  |  |  |  |  |  |  |  |  |  |
|  | Top-down process |  |  |  |  |  |  |  |  |  |  |  |
|  | Problems in agenda Setting |  |  |  |  |  |  |  |  |  |  |  |
|  | Problems of the education system ^b^ |  |  |  |  |  |  |  |  |  |  |  |
|  | Insufficient clear childhood obesity and policies effectiveness data |  |  |  |  |  |  |  |  |  |  |  |
|  | Absence of a travel plan |  |  |  |  |  |  |  |  |  |  |  |
|  | Insufficient equipment and facilities |  |  |  |  |  |  |  |  |  |  |  |
|  | insufficient staffing |  |  |  |  |  |  |  |  |  |  |  |
|  | Cycling to school is unsafe |  |  |  |  |  |  |  |  |  |  |  |
|  | Parents and administrative principals as preventing the implementation of interventions |  |  |  |  |  |  |  |  |  |  |  |
|  | Obesogenic environments |  |  |  |  |  |  |  |  |  |  |  |
|  | Limited funding and resources |  |  |  |  |  |  |  |  |  |  |  |
|  | Restrictive policies |  |  |  |  |  |  |  |  |  |  |  |
|  | Fast food and Junk food advertisement |  |  |  |  |  |  |  |  |  |  |  |
|  | Insufficient experts support of policy |  |  |  |  |  |  |  |  |  |  |  |
|  | Insufficient planning in the field of childhood obesity prevention policies |  |  |  |  |  |  |  |  |  |  |  |
|  | The emergence of not anticipated priorities |  |  |  |  |  |  |  |  |  |  |  |
|  | Unsuitable plan to cope with teacher and staff turnover |  |  |  |  |  |  |  |  |  |  |  |
|  | Develop strict policies and not implement them properly at the executive level |  |  |  |  |  |  |  |  |  |  |  |
|  | Insufficient strategy and policy guidelines clarity |  |  |  |  |  |  |  |  |  |  |  |
|  | Non-acceptance of intervention programs by parents and children and adolescents |  |  |  |  |  |  |  |  |  |  |  |
|  | Legal roadblocks |  |  |  |  |  |  |  |  |  |  |  |
|  | Magnitude of the obesity problem |  |  |  |  |  |  |  |  |  |  |  |
|  | Insufficient support for organizations and institutions outside the MoHME ^c^ |  |  |  |  |  |  |  |  |  |  |  |
|  | Low participation of stakeholders in intervention programs |  |  |  |  |  |  |  |  |  |  |  |
|  | Conflicting policies within preschools |  |  |  |  |  |  |  |  |  |  |  |
|  | Insufficient infrastructure near schools |  |  |  |  |  |  |  |  |  |  |  |
|  | Competing priorities in schools ^d^ |  |  |  |  |  |  |  |  |  |  |  |
|  | Curriculum pressure in schools |  |  |  |  |  |  |  |  |  |  |  |
|  | Limited control over food provided in schools |  |  |  |  |  |  |  |  |  |  |  |
|  | Cycling is unsuitable for girls |  |  |  |  |  |  |  |  |  |  |  |
|  | False cultural beliefs on childhood obesity |  |  |  |  |  |  |  |  |  |  |  |
|  | ***What you need to add to the list above*** |  |  |  |  |  |  |  |  |  |  |  |
| ^*^ Explanation: Individual factors mean the factors related children, adolescents, parents and executive level stakeholders  ^a^ language problem: The community language is other than language at home  ^b^ Problems of the education system in society and insufficient education for children, adolescents and parents  ^c^ Ministry of Health and Medical Education  ^d^ competing curriculum demands and priorities or competing commitments/priorities in the schools | | | | | | | | | | | | |

**Table 2.** Facilitators of childhood obesity prevention policies

| **Themes** | **Facilitators** | 1 | 2 | 3 | 4 | 5 | 6 | 7 | 8 | 9 | 10 | **Any comment** |
| --- | --- | --- | --- | --- | --- | --- | --- | --- | --- | --- | --- | --- |
| **Individual level** | User-friendliness of the intervention materials |  |  |  |  |  |  |  |  |  |  |  |
|  | ***What you need to add to the list above*** |  |  |  |  |  |  |  |  |  |  |  |
| **Executive level** | Commitment of schools staffs |  |  |  |  |  |  |  |  |  |  |  |
|  | Good relationships and teamwork with parents and school staff |  |  |  |  |  |  |  |  |  |  |  |
|  | sufficient executive staff |  |  |  |  |  |  |  |  |  |  |  |
|  | Bicultural playgroup leaders |  |  |  |  |  |  |  |  |  |  |  |
|  | Ethnic community groups |  |  |  |  |  |  |  |  |  |  |  |
|  | Strong teacher motivation |  |  |  |  |  |  |  |  |  |  |  |
|  | Taking a participatory approach to the development of program materials |  |  |  |  |  |  |  |  |  |  |  |
|  | School districts with existing collaborations |  |  |  |  |  |  |  |  |  |  |  |
|  | Effective communication between stakeholders |  |  |  |  |  |  |  |  |  |  |  |
|  | Obesity messages for the public |  |  |  |  |  |  |  |  |  |  |  |
|  | Obesity messages for policy makers |  |  |  |  |  |  |  |  |  |  |  |
|  | Obesity messages for clients |  |  |  |  |  |  |  |  |  |  |  |
|  | ***What you need to add to the list above*** |  |  |  |  |  |  |  |  |  |  |  |
| **Structural level** | Flexibility of the intervention |  |  |  |  |  |  |  |  |  |  |  |
|  | Involvement of the police in traffic workshops in the school |  |  |  |  |  |  |  |  |  |  |  |
|  | Access to feasible resources (Financial and human resources) |  |  |  |  |  |  |  |  |  |  |  |
|  | Low-cost and appropriate resources |  |  |  |  |  |  |  |  |  |  |  |
|  | Work around restrictive policies to accommodate nutrition education |  |  |  |  |  |  |  |  |  |  |  |
|  | Use of international experts |  |  |  |  |  |  |  |  |  |  |  |
|  | The availability of appropriate facilities |  |  |  |  |  |  |  |  |  |  |  |
|  | Involvement of intervention providers’ coordinator |  |  |  |  |  |  |  |  |  |  |  |
|  | Gradually introducing activities |  |  |  |  |  |  |  |  |  |  |  |
|  | Minimizing staff burden (workload) |  |  |  |  |  |  |  |  |  |  |  |
|  | Formal and informal leaders |  |  |  |  |  |  |  |  |  |  |  |
|  | Appropriate support of intervention |  |  |  |  |  |  |  |  |  |  |  |
|  | Integration of the intervention with the curriculum |  |  |  |  |  |  |  |  |  |  |  |
|  | Delivery of healthy food program in schools |  |  |  |  |  |  |  |  |  |  |  |
|  | ***What you need to add to the list above*** |  |  |  |  |  |  |  |  |  |  |  |
| ^a^ language problem: The community language is other than language at home  ^b^ competing curriculum demands and priorities or competing commitments/priorities in the schools | | | | | | | | | | | | |
