# Supplementary material for: Stakeholders perspectives of barriers and facilitators of childhood obesity prevention policies in Iran: A Delphi method study
Source: BMC Public Health. 2021 Dec 11;21:2260. doi: 10.1186/s12889-021-12282-7 (PMC8665716; doi:10.1186/s12889-021-12282-7)
Supplement: Supplementary file 3 — Additional file 3. [file 12889_2021_12282_MOESM3_ESM.pdf]

Semi-structured interview guide

Date of interview: ………………… Age: ………………….

1- Please introduce yourself

first name and last name: Job position:

How long have you been working in this job position?

Education level and degree: ………………….

Is your job related to your education?

2. What is your view on existing policies to prevent childhood and adolescent obesity, its importance and effectiveness, and similar policies?

3- What is your view on the organizational structure of childhood and adolescent obesity prevention policy in Iran?

4- In your opinion, what are the problems in the policies for the prevention of childhood and adolescent obesity in our country? (In general, related to your organization, as well as other organizations or stakeholders, and even parents and children themselves)

5. In general, what needs to be done to better implement these policies or what changes need to be made?

And other relevant items in all organizations (even if not related to your organization)

6- What is the role of your organization in the prevention of obesity in children and adolescents?

7. How is the cooperation and relationship of your organization with other institutions, organizations, institutes, etc.?

8- Do you have information about the history of the activities of your organization or other organizations about these policies? And does your organization have written guidelines for this policy?

9- Is it possible to access the relevant circulars in a part of your organization's website?

10- In your opinion, which of the underlying factors (cultural, political, social, economic) is effective in preventing childhood and adolescent obesity in Iran, and for the better implementation of this policy, which of these underlying factors and to what? How should they change?

11- In your opinion, in the prevention of childhood and adolescent obesity in Iran, which factors act as inhibitors and which factors act as facilitators?

12- In your opinion, what individuals and organizations can play an effective role in preventing childhood and adolescent obesity in Iran?

13- Please express any other point of view regarding the prevention policies of child and adolescent obesity in the country.

Telephone number: **Email:**
