# Supplementary figures and images for: Stakeholders perspectives of barriers and facilitators of childhood obesity prevention policies in Iran: A Delphi method study
Source: BMC Public Health. 2021 Dec 11;21:2260. doi: 10.1186/s12889-021-12282-7 (PMC8665716; doi:10.1186/s12889-021-12282-7)

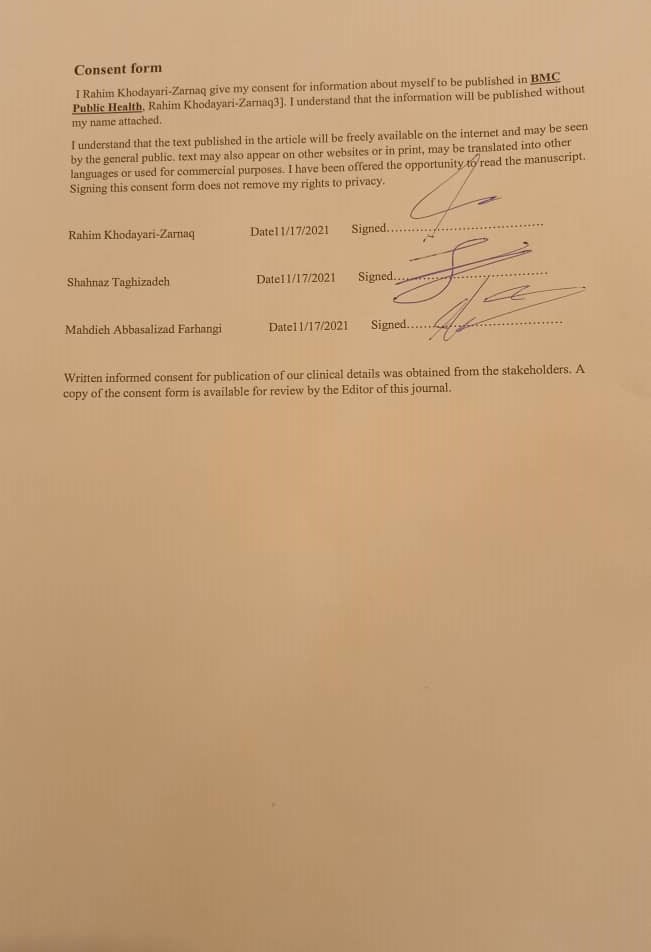

Supplement: Supplementary file 4 — Additional file 4. [file 12889_2021_12282_MOESM4_ESM.jpeg]
